# Microbiological Composition of Diets of Cactus Pear-Based with Increasing Levels of Buffel Grass Hay and Relationship to Nutritional Disorders in Sheep

**DOI:** 10.3390/ani12040500

**Published:** 2022-02-17

**Authors:** Diego de S. Vieira, Juliana S. de Oliveira, Edson M. Santos, Betina Raquel C. dos Santos, Luís Fernando B. Pinto, Anderson de M. Zanine, Diego Francisco O. Coelho, Gilberto de C. Sobral, Guilherme M. Leite, Rafael L. Soares, Francisco Naysson de S. Santos, Maria Alyne C. Santos, Nelquides B. Viana, Paulo da C. Torres Júnior, Paloma G. B. Gomes

**Affiliations:** 1 Department of Animal Science, Federal University of Paraiba, Areia 58397-000, Paraiba, Brazil; diegoosousa@live.com (D.d.S.V.); juliana@cca.ufpb.br (J.S.d.O.); edson@cca.ufpb.br (E.M.S.); cunhabrs@yahoo.com.br (B.R.C.d.S.); diego20coelho@hotmail.com (D.F.O.C.); gilbertoufrpe.zootecnia@gmail.com (G.d.C.S.); guilhermeleite33@hotmail.com (G.M.L.); rafael.lopes.soares@gmail.com (R.L.S.); alyne.123@hotmail.com (M.A.C.S.); nbv@academico.ufpb.br (N.B.V.); pauloctjunior@gmail.com (P.d.C.T.J.); pgabrielabgomes@hotmail.com (P.G.B.G.); 2 Department of Animal Science, Federal University of Bahia, Salvador 40170-110, Bahia, Brazil; luisfbp@gmail.com; 3 Department of Animal Science, Federal University of Maranhao, Chapadinha 65500-000, Maranhão, Brazil; anderson.zanine@ufma.br

**Keywords:** non-fiber carbohydrates, diarrhea, pathogenic microorganisms, ruminants

## Abstract

**Simple Summary:**

Small ruminants fed on cactus pear exclusively in the diet may present nutritional disorders, especially diarrhea. This is due to the high moisture requirements present in this food, related to microbiological factors inherent to poor hygienic quality, such as handling and prolonged exposure of crushed palm to the air, which can cause greater ingestion by animals of certain bacterial groups with pathogenic potential. The aim of this research was to investigate the levels of cactus associated with buffel grass hay on the microbiological quality of diets and the influence on physiological parameters in sheep. Thus, this research revealed that the participation of buffel grass hay was able to reduce the contamination of bacteria that cause diarrhea and depress animal performance.

**Abstract:**

This study aimed to evaluate the microbiological composition of cactus pear-based diets with increasing levels of buffel grass hay, and its effect on the blood and physiological parameters and occurrence of diarrhea in feedlot sheep. Four diets containing different percentages of buffel grass hay were tested. Diets were composed of forage cactus, buffel grass hay and concentrate, and the treatments were represented by different levels of hay in the dry matter of the feed: 7.5% buffel grass hay; 15% buffel grass hay; 30% buffel grass hay; and 45% buffel grass hay on a dry matter basis. There was a significant effect (*p* = 0.0034) of inclusion levels of buffel grass hay on fecal score. Only at the 45% inclusion level diarrhea was not observed, showing that the level of buffel grass affected more the animals than the collection period, although the collection period has affected the microbial counts. Probably there was a physiological adaptation of animals over time. There were significant changes (*p* < 0.0001) in the blood parameters of sheep. The reduction of the proportion of cactus and the inclusion of greater than 15% buffel grass hay, on a dry matter basis, provides less contamination of the diet and animal feces by enterobacteria, such as *E. coli*.

## 1. Introduction

In semiarid regions that present irregular patterns in the distribution of rainfall at a certain time of the year, the performance of animals may be limited by forage availability. One way to circumvent this limitation is to provide concentrate feed for animals, and/or use forage plants adapted to climatic and soil conditions, such as cactus pear [1,2].

In this sense, the cactus pear is a plant with high adaptability and resistance to weather and that is present in several semiarid regions of the world.

As a food, the cactus pear is an excellent energy source, due to its high content of non-fiber carbohydrates, and is an excellent source of water, due to its moisture content [2]. Thus, the tendency of farms in the semiarid region is to use as much cactus pear as possible in rations.

In turn, the exclusive use of cactus pear in animal diets, can cause nutritional disorders to animals, such as diarrhea, foamy bloat and ruminal acidosis [3]. Several authors relate these nutritional disorders to low levels of physically effective neutral detergent fiber (NDFpe) in cactus pear [4,5]. However, the appearance of nutritional disorders in ruminants fed high levels of cactus pear is also related to microbiological factors regarding poor hygienic quality, such as handling and exposure of crushed cactus pear to air for a long time, which can provide a greater intake by animals of certain bacterial groups with pathogenic potential, for example, the order *Enterobacteriales*, responsible for diarrhea [4]. Considering that cactus pear is rich in water, when it is chopped and exposed to air for hours during the animal feeding it could allow microorganisms access to moisture and nutrients, leading to a microbial contamination.

Thus, it is important to use other sources fiber sources from hay or forage in diets based on cactus pear, as it implies in reducing the incidence of nutritional disorders not only by improving the NDFpe of the diet, but possibly also by reducing both moisture and soluble carbohydrate content [5].

Thus, buffel grass (*Cenchrus ciliaris* L.) can be an alternative, as it is one of the most cultivated grasses in arid and semiarid regions, mainly due to its tolerance and adaptability to low rainfall [6]. Among the ways to use the buffel grass, that the grass has favorable characteristics to be preserved as hay, as it has a high leaf:stem ratio, thin stems and narrow cuticle.

However, although there are several studies on the combination of buffel grass hay with cactus pear, aiming to improve animal performance, there are no studies on the relationship between nutritional disorders and the growth of microorganisms with pathogenic potential in diets with cactus pear.

In this context, this study aimed to evaluate the microbiological composition of cactus pear-based diets with increasing levels of buffel grass hay, and its effect on the blood and physiological parameters and occurrence of diarrhea in feedlot sheep.

## 2. Materials and Methods

### 2.1. Place and Period of Execution of the Experiment

The experiment was conducted on a private property located in the municipality of São José dos Cordeiros, between September and November 2020.

The municipality of São José dos Cordeiros is located in the central region of the state of Paraíba, Borborema Mesoregion and Western Cariri Microregion, with latitude: 7°23′26″ S, Longitude: 36°48′30″ W and 529 m altitude. The climate of the region is classified as semiarid BSh, according to the Köppen classification, with average rainfall of 551.0 mm per year.

### 2.2. Animal Management and Experimental Treatments

For the experimental trial, 40 rams with non-descript breed with an initial body weight of 18 kg ± 2.6 kg were used. In the period before the experiment, animals were weighed, vaccinated against Clostridiosis and treated against endo- and ectoparasites. Animals were housed in individual pens, measuring approximately 3 m^2^, with a feeding trough to supply the diet and a drinking fountain with unrestricted access to water. The experiment lasted 60 days, divided into ten days for the pre-experimental period for adaptation to the facilities and diets, and 50 days for data collection. Rations were based on cactus pear “Mexican Elephant Ear” (*Opuntia stricta* Haw) and buffel grass hay (*Cenchrus ciliaris* L.), both harvested one year after planting (Table 1).

Animals were distributed in a completely randomized design, with four treatments and 10 replications. Four diets consisting of different levels of inclusion of buffel grass hay in the dry matter of the feed were evaluated: 7.5% FB = 7.5% buffel grass hay; 15% FB = 15% buffel grass hay; 30% FB = 30% buffel grass hay; and 45% FB = 45% buffel grass hay on a dry matter (DM) basis. Diets were formulated to be isonitrogenous, and meet the requirements, according to the [7], of sheep with an average weight of 18 kg, for a weight gain of 200 g/day (Table 2).

### 2.3. Food Management and Consumption

Feed was supplied ad libitum in two equal daily portions, at 08:00 and 16:00 h, weighed and adjusted, allowing 10% leftovers. Leftovers were daily weighed to control the intake of dry matter and other nutrients by the animals. Leftovers were collected before supplying the feed, both in the morning and in the afternoon.

Dry matter (DM) intake occurred with the daily weighing of the diet offered and the leftovers between days 15 and 49 of the experimental period. Dry matter intake (DMI) was calculated from the difference between the ingested amount of feed and leftovers, both on a DM basis. For this, samples of food, diets and leftovers were collected every seven days of the experimental period, to determine the dry matter content.

### 2.4. Food Sampling and Analysis

Chemical analyses were performed on samples of ingredients and feeds in the food analysis laboratory of the Semiarid National Institute (INSA), Campina Grande, state of Paraíba. For this purpose, samples were analyzed for dry matter (DM; method 934.01), mineral matter (MM; method 942.05), crude protein (CP; method 954.01), ether extract (EE; method 920.39) and lignin (method 973.18) were determined, according to [8]. Neutral detergent fiber (NDF) and acid detergent fiber (ADF) were determined according to the methodology proposed by [9], using the ANKOM fiber analyzer (ANKOM200 Fiber Analyzer—ANKOM Technology Corporation, Fairport, NY, USA).

The non-fiber carbohydrate (NFC) content of ingredients and feed was calculated using the equations recommended by [10]: = 100 − (%CP + %NDFcp + %EE + %MM).

### 2.5. Blood Collections, Serum and Physiological Parameters

Blood was collected from all sheep by puncturing the jugular vein using Vacutainer tubes containing EDTA (ethylenediaminetetraacetic acid) (0.1 mL 10% EDTA) with a volume of 5 mL each, on the 1st, 6th and 26th day of the experimental period. Collections were carried out before the morning meal and sent immediately to the laboratory for complete blood count and identification of microangiopathic hemolytic anemia due to verotoxins, considering as standard values of the experiment those found in the blood analyzed in the 1st day of the experimental period.

The blood count evaluated: Hemoglobin (Hb) (cyanomet hemoglobin described by [11], Mean corpuscular volume (MCV), Mean globular hemoglobin concentration (CHCM), Platelets, Total plasma protein (TPP) by refractometry, Fibrinogen (FP), Leukocytes (hemocytometer), Rods, Segmented, eosinophils and monocytes [12]

On the second and 22nd of the experimental period, at 9:00 am, the physiological variables of all animals were collected. The respiratory rate was determined by counting the flank movements for 15 s, this result was multiplied by four to obtain the number of respiratory movements per minute. Rectal temperature was measured using a clinical thermometer inserted into the rectum for one minute, with individual readings, followed by notes. Skin surface temperatures on the nape, shoulder, leg and belly were also measured using an infrared thermometer, at a distance of 30 cm from the animal’s surface, directed transversely to the specific location.

### 2.6. Fecal Score and Microbial Population Count

The fecal score of the animals was determined according to consistency, which varies from 0 (normal consistency) to 4 (watery consistency), according to the [13].

On the 1st and 21st day of the experimental period, samples were taken from feces (directly from the rectum) and leftovers (removed from the troughs) for microbiological evaluation, in which the growth of enterobacteriaceae and *E. coli* (fecal contamination indicator). For this, samples were taken and immediately sent to the laboratory in a Styrofoam box with ice.

To evaluate the microbial populations, 25 g feces and dietary leftovers were collected in the trough, added in two hundred and twenty-five milliliters (225 mL) of 0.1% sterile buffered peptone water solution. Through the selective technique of culture medias, violet-red bile lactose agar was used for growth, for the cultivation of enterobacteriaceae and E.M.B Levine agar for the cultivation of *E. coli*. From the first dilution, new serial dilutions were made, the material from each treatment was grown in sterile Petri dishes using the pour-plate method with the respective culture media, in duplicate, and incubated in a bacteriological incubator at 36 °C for 24 h.

Plates for counting were those with values between 30 and 300 CFU (colony forming units), considering the dilution. Colonies of enterobacteria showed a circular shape and pink coloration, and *E. coli* showed a similar shape, but with a metallic green color.

### 2.7. Statistical Analysis

#### 2.7.1. Blood, Microbiological Count and Physiological Parameters

The means of the different periods were compared by Tukey’s Test using the SAS^®^ software. A 5% significance level was used in all hypothesis tests. Given the correlation between the values obtained from the same animal in different periods of evaluation, analysis of variance with repeated measures over time was used. The choice of the covariance matrix for this analysis was made from the analysis of different matrices and the matrix that resulted in the lowest BIC (Bayesian Information Criterion) was selected. When the effect of treatment was significant, their degrees of freedom were broken down into linear, quadratic and cubic contrasts. A mixed model was used, which can be described as

Yijkl = μ + Ai + Xj + Pk + (XP) + jk + εijkl(1)

where Yijkl is the record of the variable of interest; Ai is the random effect of the i-th animal, where i = 38 levels in the analysis of blood count data and i = 20 levels in the analysis of micobiology data; Xj is the fixed effect of the jth level of buffel grass hay, where j = (1,…,4), Pk is the fixed effect of the kth period, where for hemogram variables k = (1,…,3), while for microbiology variables k = (1,2); (XP) jk is the fixed effect of the interaction between the jth buffel grass hay and the kth period; and εijkl is the random effect of the residual.

#### 2.7.2. Fecal Score

For the analysis of fecal score, as it is a discrete ordinal variable, it was decided to implement a generalized linear model. This model can be described as

Yijk = μ + Xi + Pj + (XP) + ij + εijk
(2)

where Yijk is the fecal score record; Xi is the fixed effect of the i-th level of buffel grass hay; Pj is the fixed effect of the j-th period; (XP) ij is the fixed effect of the interaction between the i-th% buffel grass hay and the j-th period; and εijk is the random effect of the residual. This model was fitted with a multinomial distribution and the CUMLOGIT option as a link. In this analysis, 5% was also assumed as the significance level.

## 3. Results

**Figure 1:**
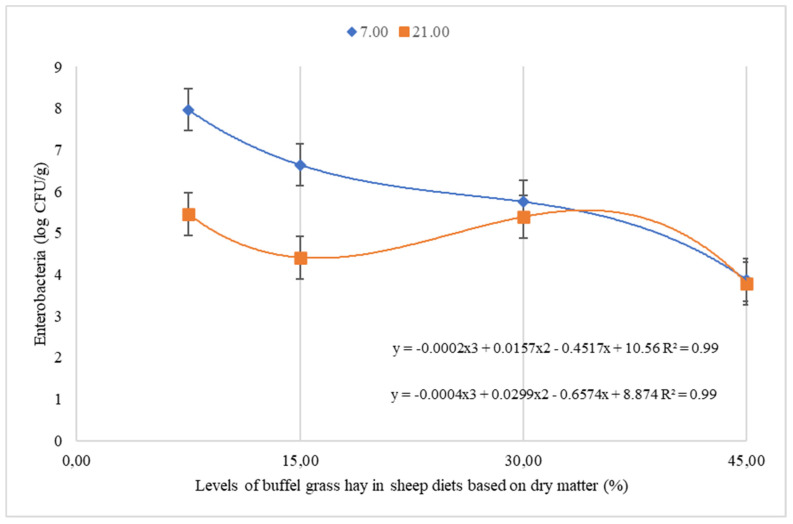
Growth of Enterobacteriaceae in the leftovers of sheep fed levels of buffel grass hay and cactus pear. CFU = colony forming unit. 7.00 = 7th day and 21.00 = 21st day.

**Figure 2:**
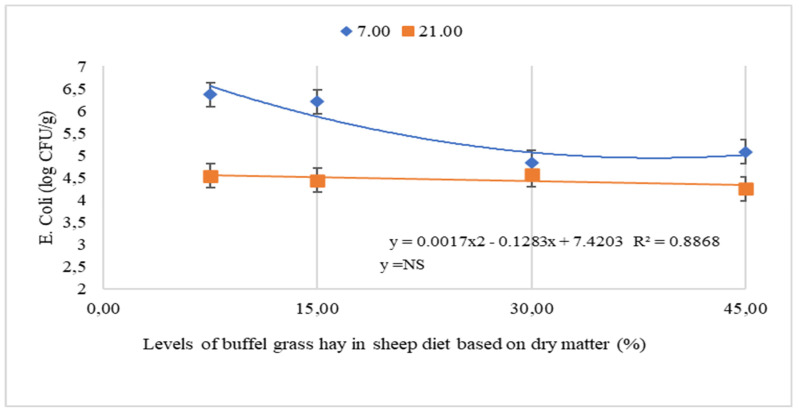
Growth of Escherichia coli in the leftovers of sheep fed levels of buffel grass hay and cactus pear. CFU = colony forming unit. 7.00 = 7th day and 21.00 = 21st day.

An effect of interaction of collection period and buffel grass hay levels was detected on the counts of Enterobacteriaceae (*p* < 0.0001) and *E. coli* (*p* = 0.0043) (Table 3). Both on the 1st and 21st day, there was a cubic effect of the levels of buffel grass hay on the count of Enterobacteriaceae in the leftovers (Figure 1). There was also a quadratic effect of buffel grass hay levels on *E. coli* count on day 1 (Figure 2). The lowest *E. coli* count in the leftovers occurred when the animals consumed 32.7% buffel grass hay (Figure 2). There was no significant difference in the *E. coli* count in the leftovers on the 21st day, with a mean value of 4.45 log CFU/g.

On the first day of collection, leftovers of animals consuming the lowest levels of buffel grass hay (7.5% and 15%) had the highest count values for both Enterobacteriaceae (7.97 log CFU/g and 6.37 log CFU/g) and *E. coli* (6.64 log CFU/g and 6.21 log CFU/g) compared to the 21st day, with values of 5.46 log CFU/g for Enterobacteriaceae and 4.54 log CFU/g for *E. coli* (7.5% buffel hay) and 4.41 log CFU/g for Enterobacteriaceae and 4.44 log CFU/g for *E. coli* (15% buffel hay). The counts in the leftovers of animals consuming 30% and 45% buffel grass hay did not differ between collection periods, with mean values of 5.57 log CFU/g and 3.83 log CFU/g for Enterobacteriaceae and 4.71 log CFU/g and 4.66 log CFU/g for *E. coli*, respectively.

As for feces, there was a significant difference (*p* < 0.0001) in the counts of Enterobacteriaceae and *E. coli* (Table 3). The highest counts were found in the 1st collection period for both Enterobacteriaceae (8.01 log CFU/g) and *E. coli* (7.06 log CFU/g). There was no effect of diets on the growth of Enterobacteriaceae and *E. coli* in animal feces, with mean values of 6.72 log CFU/g and 5.73 log CFU/g, respectively.

There was no effect of interaction (*p* = 0.9311) on the fecal score of the animals (Table 4). However, there was a significant effect (*p* = 0.0034) of the levels of buffel grass hay on this variable (Table 4). Animals fed 7.5% and 15% buffel grass hay presented the highest probabilities of score two, with averages of 70.7% and 63.2%, respectively. Animals fed 30% buffel grass hay had higher probability values in score one. Animals fed 45% buffel grass hay scored zero in 100% samples.

The quadratic effect of the levels of buffel grass hay on the dry matter intake of the animals is observed, in which the maximum consumption of sheep is estimated at the level of 25.3% of buffel grass hay (Table 4).

There was no effect on the physiological variables: rectal temperature (*p* = 0.953), nape temperature (*p* = 0.263), shoulder temperature (*p* = 0.246), leg temperature (*p* = 0.257), and heart rate (*p* = 0.496). Probably, there was an adaptation of the animals to a high load of microorganisms with pathogenic potential (Table 5), which can be proven by the non-significance of the levels of buffel grass hay and the period on most of the physiological parameters of the animals (Table 5).

There was no effect of buffel grass hay levels in the diet and collection day on mean corpuscular volume (VHCM) and platelets, with mean values of 30.5 fL and 776,070.75 μL, respectively (Table 6).

There was an effect of the collection period (*p* < 0.0001). On the 21st day (9.45 × 10^6^ mm^3^), there was a higher concentration of erythrocytes than on the 1st day (7.33 × 10^6^ mm^3^) and on the 7th day (7.90 × 10^6^ mm^3^). However, there was no effect of buffel grass hay levels on erythrocyte concentrations (*p* = 0.4139) with a mean value of 8.22 × 10^6^ mm^3^.

Hemoglobin values had a significant effect (*p* < 0.0001) for the collection periods and there was an effect of interaction of hay levels with collection periods (*p* < 0.0001). Animals consuming 7.5% buffel grass hay had higher hemoglobin concentrations on the 21st day (9.24 g/dL and 9.43 g/dL) than on the 1st day (7.17 g/dL and 8.10 g/dL). Animals consuming 15% and 30% buffel grass hay had higher hemoglobin concentration on the 21st day. There was no effect of period on the hemoglobin concentration in animals fed 45% buffel grass hay, with a mean value of 8.26 g/dL.

Hematocrit values showed a significant difference (*p* < 0.0001) for the collection periods, with an increase in the amount of hematocrit on the 21st day compared to the 1st and 7th day (Table 6).

There was a significant effect (*p* < 0.0001) for the collection periods and interaction of buffel grass hay levels with collection periods (*p* = 0.0092) on the mean corpuscular hemoglobin concentration (CHCM). Mean values of CHCM of animals fed 7.5% buffel on the 1st day (30.18 g/dL) were lower than the 7th (33.07 g/dL) and 21st (32.90 g/dL) days. With respect to animals fed the other levels of buffel grass hay, there was no effect of period on this variable.

There was a significant effect (*p* = 0.0106) for the collection periods and interaction of the levels of buffel grass hay with the collection periods (*p* = 0.0090) for leukocytes (Table 6). Animals receiving 15% buffel grass hay had higher concentrations of leukocytes on the 7th (8011.11 μL) and 21st (7955.56 μL) days when compared to the same animals on the 1st (5277.78 μL) day of collection. There was no effect of period on leukocytes of animals consuming 7.5; 30 and 45% buffel grass hay, with mean values of 7349.73 μL; 7185.19 μL and 8769.58 μL, respectively.

The values of segmented showed significant differences for the collection period (*p* < 0.0001) and interaction (*p* = 0.0083). Animals consuming 15% buffel grass hay had higher segmented values on the 7th day (8011.11 μL) and 21st day (7955.56 μL) when compared to the 1st day (5277.78 μL). Animals fed 45% buffel grass hay had higher segmented values on the 7th day when compared to the 21st day. Animals receiving 7.5 and 30% buffel grass hay had similar segmented values in the different collection days.

There was interaction (*p* < 0.0001) on eosinophils, and animals receiving 30% on the 21st day had the highest blood eosinophil levels (Table 6). Animals from other treatments had similar eosinophil values on the 1st, 7th and 21st day. There was also a quadratic effect of buffel grass hay levels on eosinophil values on the 1st day and cubic effect on the 7th and 21st days.

**Figure 3:**
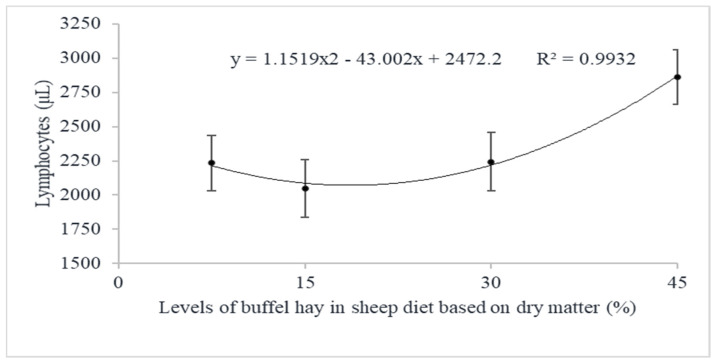
Effect of levels of buffel grass hay on lymphocytes.

There was no effect of interaction of buffel grass hay levels with period in relation to lymphocytes. However, there was a quadratic effect of levels of buffel grass hay on this parameter, with a minimum value of 18.7% buffel grass hay (Figure 3). There was also an effect of period on lymphocytes, in which there was a lower concentration of lymphocytes on the 7th day when compared to the 1st and 21st days.

There was an interaction (*p* < 0.0001) for monocytes (Table 6). On the 1st day of collection, animals consuming 7.5% buffel grass hay had lower monocyte values 217.8 μL) when compared to the 21st day (554.40 μL). In relation to the animals consuming 15% buffel grass hay, on the 7th day, the animals had higher values than on the 1st day. Animals fed 30% and 45% buffel grass hay showed no significant difference in monocytes between the collection periods.

Values of total plasma proteins showed interaction of buffel grass hay levels with collection periods. The highest concentration was found in animals consuming 15% buffel grass hay, on the 7th day of collection (7.52 g/dL).

## 4. Discussion

It is possible to infer that the growth of both Enterobacteriaceae and *E. coli* as reduced with the lowest proportions of cactus in the diets. At the level of 7.5% of buffel grass hay, there was a greater proliferation of these microorganisms, possibly due to the high moisture content in the diet. The moisture content is a factor that improves microbial growth, as it must be considered when the food is chopped and offered to the animals, since it can be exposed to the air for several hours, providing an ideal environment for development. of pathogenic microorganisms such as Enterobacteriaceae, including *E. coli*. Cactus pear is a food with high moisture content and its composition is rich in substrates for microbial fermentation, such as soluble carbohydrates. When cut into smaller structures, there is a greater surface area of the forage cactus available for access to nutrients by microorganisms, allowing for greater growth and fermentation [4].

This may have occurred due to the increase in the pH of the diet, as well as the greater exposure of the material to oxygen, when reducing the amount of cactus in the diet provided. However, the increase in the proportion of buffel grass hay, which has a more alkaline pH [14], combined with cactus pear mucilage that has as a buffer effect [15], in combination, may have created a suitable environment for the growth of these microorganisms. In addition, water in food can form a physical barrier for oxygen to enter material exposed to air, so the drier the food, the more easily air can penetrate it.

The interaction of high moisture content of the diet with 7.5% cactus pear, particles smaller than 2 cm^2^, and time of exposure to air in the diet, proved to be the combination that provides the highest counts of Enterobacteriaceae and *E. coli* in leftovers, which can lead to deterioration of the material as well as ingestion of foods with a high concentration of agents with the potential to harm the animal health.

*E. coli* is the most common species in the intestines, and when found in water and food, it indicates fecal contamination, which can be pathogenic and cause diseases [16]. Very high counts may indicate the ingestion of contaminated food that culminates in the infection of the animal organism, causing diarrhea, intense fever and dehydration due to watery feces [4]. Probably, the processing of cactus before feeding the animal and stored in an environment with poor hygiene stimulates the growth of enterobacteria, reflecting in a diet with a higher bacterial count, therefore, high amounts of these bacteria can be excreted in the feces. Enterobacteriaceae are commonly found in the most diverse environments and in animals feces, thus poor hygiene environment could result in contamination of the rich cactus diets when feeding the animals, resulting in infections and diarrhea.

The lower count of these microorganisms in the leftovers on the 21st day in diets containing 7.5 and 45% buffel grass hay compared to the 1st day of the experimental period is probably due to the adaptation of the animals over the days to this high concentration of microorganisms.

The presence of Enterobacteriaceae and *E. coli* was expected since these microbial groups are present in the animal organism [16]. The microbiological counts of Enterobacteriaceae on the first experimental day are similar to the values reported by [4], who observed a count of 8.48 CFU/g in the diet with crushed cactus pear eight hours before feeding. In a biochemical profile test, the presence of *E. coli* in the feces of animals was confirmed, reinforcing that these microorganisms inhabit the gastrointestinal tract of sheep.

The highest count of Enterobacteriaceae and *E. coli* in feces of the animals on the first day of collection was possibly because the animals were still adapting to the levels of cactus pear in the diets, and it may be a consequence of the possible ingestion of a food with a higher bacterial load, given the higher count of these microorganisms in food leftovers, also on the first day of collection. However, throughout the experimental period, the animals become adapted to their diets, promoting a balance between the microorganisms present in the gastrointestinal tract of sheep, as the counts of Enterobacteriaceae and *E. coli* in feces were lower on the 21st day (Table 3).

Changes in the fecal score of sheep are related to the amount of cactus pear used in this study. The higher probability of fecal score two in animals fed 7.5% and 15% buffel grass hay is likely due to the lower DM content of these diets (Table 3). The animals had higher counts of Enterobacteriaceae and *E. coli* only on the first day of collection for these levels (Table 4) which suggest that that it was an effect of moisture on the microbial counts.

When cactus pear is offered in too high amounts in the ruminant diet and without the provision of an extra source of forage, changes in the characteristics of animal feces are observed, generally less solid than normal, due to the increased passage rate caused by the high amount of water available and low fiber content in the diet based on cactus pear [17].

Therefore, the opposite should also be taken into account, since the higher levels of fiber in the diet for ruminants can reduce the moisture content and regulate the passage rate through the gastrointestinal tract, also affecting the growth of Enterobacteriaceae and *E. coli* in the feed (Table 4), which interferes with the microbiota of the gastrointestinal tract and, consequently, with the occurrence of diarrhea in the animals (Table 4). As a consequence, the level of 45% buffel grass hay presented a fecal score equal to zero, which is considered the normal value for sheep.

There is a quadratic effect of the levels of buffel grass hay on the dry matter intake of the animals, in which the maximum intake of sheep is estimated at the level of 25.3% buffel grass hay (Table 4). The reduction in intake within the range of tested levels (7.5 and 45% FB) may be an adaptation of the animal to food with a high microbial load. Contaminated feed tends to reduce the intake in ruminants as this can cause negative effects on ruminal fermentation affecting the performance and health of animals [18].

A reduction in the dry matter intake of animals receiving diets with the highest counts of Enterobacteriaceae and *E. coli* was also observed [4]. Therefore, it can be assumed that the animals tended to consume a smaller amount of food due to its greater contamination, as a defense mechanism of the animal itself.

When ruminants are fed low-fiber diets, their ruminal pH drops, their microbial ecology changes, and they become more prone to metabolic disturbs [16]. Evaluating the effect of dietary fiber level in relation to dry matter (DM) intake of feedlot sheep, [19] observed that DM intake increased linearly as dietary fiber levels increased from 8.67% to a maximum of 34.69%. In the present study, dietary fiber levels were also increased according to the levels of buffel grass hay (Table 2) with DMI maximization with 25.3% buffel grass hay, above that, there was a gradual reduction in the DMI with the inclusion of this forage (Table 4). Considering that the 15% buffel grass hay diet contained 36.34% NDF and the 30% buffel grass hay diet contained 43.92% NDF (Table 3), the estimated dietary NDF value that would maximize the DMI was close to the cited authors. In addition to the high dietary pH, which may have negatively influenced the microbiological quality of the diet, as discussed above, excess fiber can also depress the animal’s DMI due to the filling effect [19].

There was an effect of interaction (*p* < 0.0001) on the belly temperature of the animals. However, this effect does not allow the inference of any pathological change in the animals, since, of the physiological parameters analyzed, rectal temperature and respiratory rate are the parameters that could indicate some type of anomaly in thermal comfort or in the health of the animals [20]. Nevertheless, the mean values of these parameters are in accordance with the reference values used by [21] for Dorper sheep that have been naturalized or crossed with native sheep, under tropical conditions, which are 38.3–39.9 °C and 20–34 moves per minute, respectively. These results reinforce that the animals get adapted to a high load of potentially pathogenic microorganisms.

The way of managing diets can positively influence, favoring the animal adaptation to diets with microbiological risk, such as cactus pear. In the present study, cactus pear was crushed and immediately offered to the animals, and the leftovers were removed twice a day, with cleaning of the troughs before supplying a new meal. Studies prove that in diets with high levels of cactus pear, the provision of cactus pear to the animals, immediately after grinding, contributes to a significant drop in Enterobacteriaceae counts in sheep feces, as reported by [4]. Thus, the way of managing and using cactus pear in diets with high levels of this plant in its composition can interfere with the growth of microorganisms with pathogenic potential, which may be correlated with cases of nutritional disorders or reduced performance of sheep confined.

In the present study, an effect of interaction (*p* < 0.0001) was detected for the hemoglobin data, which were lower on the first day and higher in animals fed 45% buffel grass hay. Moreover, hematocrit values showed significant differences between collection periods, which may reinforce some anomaly in animal health (possible anemia) during the first days of the experimental period and a possible adaptation by these animals over the days. Thus, the results of blood parameters allowed inferring that the animals were able to adapt to the diets even with the high levels of cactus pear in its composition.

The mean values of erythrocytes, hemoglobin and hematocrit are close to those reported by [4,22]. Reference [4] concluded that animals can adapt to diets with high levels of cactus pear depending on the way of feed management. This study reinforces our findings, as it was carried out under similar conditions and with animals with the same genotype and phenotype patterns.

In the present study, animals fed 7.5% buffel grass hay had lower hemoglobin concentration values on the first day of collection, which would be explained by the fact that the animals are still adapting to their diets (Table 6). However, it is noteworthy that despite the lower means presented by the level of 7.5% buffel grass hay on the 1st day of collection, these values were within the reference range suggested in the complete blood count exam (30–36 g/dL) and remained both in the second (33.07 g/dL) and in the third collection (32.90 g/dL).

Values of total leukocytes are responsible for indicating a possible infection in the animal organism, whether caused by viruses or bacteria, in suspected blood diseases, parasitic infections or allergic processes [23]. In the present study, values of total leukocytes at 15% buffel grass hay were lower on the first day of collection compared to the 7th and 21st days, which may indicate an immune response of the animal to a possible infection caused by ingestion of foods with higher counts of Enterobacteriaceae and *E. coli* in the first days of the experimental period (Table 3). The highest counts of Enterobacteriaceae and *E. coli* in the animals’ feces on the first day of collection coincide with the period of blood collection, in which the animals had the lowest total leukocyte counts, so it is possible to state that the microorganisms affected the health of the animals. Values of total leukocytes below those indicated may represent a condition of leukopenia, where severe cases suggest infections [23]. However, the total leukocyte counts on the 1st day of collection was 5277.78 μL, being within the reference range indicated by the complete blood count test, which is 4000–12,000 μL, indicating that despite the drop, the animals did not progress to more severe conditions and over the days, they managed to recover, through the immune system, because in the 2nd collection period (7th day) the concentration of total leukocytes increased (Table 6).

The increase in leukocytes indicates that there was a possible inflammatory or infectious process occurring in the organism. The reduction in the counts of Enterobacteriaceae and *E. coli* on the 21st day of the experimental period also demonstrates that the animals were able to recover from a possible infection over the experimental days, without major complications. Values of total leukocytes on the 21st day, in animals fed 15% buffel grass hay, were similar to those found for healthy male Dorper sheep [24] which was 7576.0 μL, reinforcing that there was recovery of the animals over the days.

Values of segmented at the 15% inclusion level of buffel grass hay, on the first day of collection, may be related to the values of total leukocytes indicating the presence of an infection caused by bacteria. On the 7th day, the highest values indicate the immune response of the organisms and on the 21st day, the values reduced to a value that is in accordance with the reference literature [6,24].

Some blood parameters of the animals were affected by the levels of buffel grass hay in the diet with inclusion of cactus pear. Most of them may be related to the highest total leukocyte count, since the lowest count was found at the level of 15% buffel grass hay inclusion on the 1st day of collection, and the highest on the 7th day of collection, with also the lowest (1st day) and highest (7th day) values for segmented, eosinophils, monocytes and plasma proteins. This indicates recovery by the animal organism from a possible bacterial infection caused by lower levels of buffel grass hay in the diet and higher concentrations of cactus pear.

Despite the pasty consistency presented by the feces of animals receiving 7.5% and 15% buffel grass hay, no cases of severe digestive disorders, such as totally liquid feces or even with the presence of blood were observed. Probably, defenses of the animal organism managed to prevent the appearance of diseases even at certain levels of food contamination. Thus, it can be stated that sheep become adapted to diets with high content of cactus pear and low fiber content from buffel grass hay, even those with higher counts of Enterobacteriaceae and *E. coli*.

Thus, high levels of cactus pear in the sheep diet alter the microbiological, blood and fecal parameters of confined sheep in the first periods of diet allowance, however, the animals manage to adapt to the diets without major complications.

The research results demonstrate that the cause of nutritional disorders in ruminants consuming diets with a high proportion of cactus pear is not exclusively due to the low fiber content of physically effective diets. The high concentration of non-fiber carbohydrates in fresh cactus pear, and the inadequate feeding management of this forage, can predispose the animals to contamination by pathogenic microorganisms that will negatively interfere with the animal health, causing, among other problems, diarrhea. As forages have a low content of NFC, in the form of hay, they contain low moisture, reduce the proliferation of microorganisms in diets with cactus pear, by decreasing the favorable environmental characteristics for the growth of these microorganisms.

Nevertheless, it was also observed that sheep were able to adapt to diets with low levels of buffel grass hay. Thus, producers, especially in semiarid regions, can use cactus pear as a basis for the diet of confined sheep, provided it is correctly managed, so that the multiplication and ingestion of undesirable and possibly pathogenic microorganisms is avoided, for favoring secondary fermentations in the feeding trough of the animals.

Although physically effective fiber levels help to reduce nutritional disorders in sheep fed diets with high levels of cactus pear, the inadequate management of this food can also cause nutritional disorders. However, studies on the subject are still scarce, suggesting the need for more research to obtain more precise results.

## 5. Conclusions

Reducing the proportion of palm and adding more than 15% buffel grass on a dry matter basis provides less contamination of the diet and animal feces by enterobacteria, such as *E. coli*, without any negative change in blood parameters and reducing the occurrence of diarrhea in confined sheep.

## Figures and Tables

**Table 1:** Chemical composition of ingredients used in the experimental diets on a dry matter basis.

	Ingredients					
Item ^1^ (g/kg DM)	Cactus	Buffel Grass Hay	Corn Bran	Soybean Meal	Cotton Pie	Urea
DM ^2^	123.0	913.5	892.4	906.0	927.0	975.3
CP	44.0	38.3	94.7	400.0	202.7	281.0
NFC	518.9	50.5	647.8	342.3	148.5	0.0
EE	19.3	10.8	66.0	17.1	94.3	0.0
MM	118.0	79.2	16.4	64.8	46.8	2.1
NDF	299.8	821.2	175.1	175.8	507.7	0.0
ADF	184.2	486.0	38.9	86.6	276.8	0.0
Cellulose	141.3	376.1	34.3	81.8	245.5	0.0
Hemicellulose	115.1	302.8	90.0	69.3	207.3	0.0
Lignin	45.4	79.7	12.1	15.6	41.8	0.0

^1^ DM = Dry Matter; CP = Crude protein; NFC = Non-fibrous carbohydrates; EE = Ethereal extract; MM; Mineral matter; NDF= Neutral detergent fiber; ADF = Acid detergent fiber. ^2^ based on natural matter.

**Table 2:** Proportions of ingredients and chemical composition of experimental diets for sheep fed different levels of buffel grass hay, on a dry matter basis.

	**Diets ^1^**			
**Item (g/kg DM)**	**7.5%**	**15%**	**30%**	**45%**
Cactus pear	409.3	336.6	191.2	45.8
Buffel grass hay	72.7	145.4	290.8	436.2
Soybean meal	64.6	64.6	64.6	64.6
Corn bran	235.6	235.6	235.6	235.6
Cotton pie	178.4	178.3	178.1	178.0
Urea	8.8	8.9	9.0	9.2
Mineral nucleus	16.2	16.2	16.2	16.2
Ammonium chloride	13.5	13.5	13.5	13.5
Ammonium sulfate	1.0	1.0	1.0	1.0
**Item (g/kg DM)**	**Chemical Composition ^2^**			
DM ^3^	248.7	285.6	406.2	703.0
CP	141.3	142.0	141.7	142.3
NFC	442.0	409.9	347.0	283.4
EE	42.2	41.5	40.3	39.0
MM	48.9	43.2	31.8	20.4
NDF	325.6	363.4	439.2	514.9

^1^ 7.5% Buffel grass hay; 15% Buffel hay; 30% Buffel hay; 45% Buffel hay based on dry matter; ^2^ DM = Dry Matter; CP = Crude protein NFC = Non-fibrous carbohydrates; EE = Ethereal extract; MM = Mineral matter; NDF = Neutral detergent fiber. ^3^ Based on natural matter.

**Table 3:** Bacteria count in leftovers and feces of sheep fed levels of buffel grass hay and cactus pear.

Log CFU/g ^1^	Levels Buffel Grass Hay %	Period (Days)		Overall Average (BH)	F ^2^ Test *p*-Value			*p*-Value		
		1°	21°		BH	P	Interaction (BH x P)	%BH ^3^		
								L	Q	C
Enterobacteria, Leftovers	7.5	7.97 a ± 0.51	5.46 b ± 0.51	6.72 ± 0.49	0.0052	<0.0001	<0.0001	0.3670	0.0002	0.0077
	15	6.64 a ± 0.51	4.41 b ± 0.51	6.02 ± 0.49				0.5414	0.1173	0.0498
	30	5.75 a ± 0.51	5.39 a ± 0.51	5.57 ± 0.49						
	45	3.87 a ± 0.51	3.79 a ± 0.51	3.83 ± 0.49						
	Overall Average (P)	6.06 ± 0.25	5.01 ± 0.25							
*E. coli*, Leftovers	7.5	6.37 a ± 0.27	4.54 b ± 0.27	5.46 ± 0.23	0.0502	<0.0001	0.0043	0.5584	0.0002	0.6641
	15	6.21 a ± 0.27	4.44 b ± 0.27	5.33 ± 0.23				0.9881	0.7555	0.4027
	30	4.84 a ± 0.27	4.57 a ± 0.27	4.71 ± 0.23						
	45	5.08 a ± 0.27	4.25 a ± 0.27	4.66 ± 0.23						
	Overall Average (P)	5.63 ± 0.14	4.45 ± 0.14							
Enterobacteria, Feces	7.5	8.24 ± 0.19	5.41 ± 0.19	6.83 ± 0.15	0.7663	<0.0001	0.3328	0.7191	0.3344	0.8749
	15	8.03 ± 0.19	5.50 ± 0.19	6.76 ± 0.15						
	30	7.73 ± 0.19	5.50 ± 0.19	6.62 ± 0.15						
	45	8.03 ± 0.19	5.32 ± 0.19	6.67 ± 0.15						
	Overall Average (P)	8.01 a ± 0.09	5.43 b ± 0.09							
*E. coli*, Feces	7.5	6.73 ± 0.22	4.76 ± 0.22	5.71 ± 0.15	0.5140	<0.0001	<0.1243	0.8374	0.3487	0.2521
	15	7.10 ± 0.22	4.32 ± 0.22	6.00 ± 0.15						
	30	7.17 ± 0.22	4.83 ± 0.22	5.74 ± 0.15						
	45	7.25 ± 0.22	4.24 ± 0.22	5.75 ± 0.15						
	Overall Average (P)	7.06 a ± 0.11	4.54 b ± 0.11							

^1^ CFU = Colony forming units; ^2^ BH = Buffel grass hay; P = collection periods; ^3^ L = Linear, Q = quadratic and C = cubic; 5% significance level. The means followed by different letters on the same line differ from each other by the Tukey test at 5% significance.However, Table 3 and Figure 1 and Figure 2 show that in leftovers from animals consuming 45% buffel grass hay, there was greater growth of Enterobacteriaceae and *E. coli* than in leftovers from animals consuming intermediate levels of buffel grass hay (15 and 30%).

**Table 4:** Dry matter intake (DMI) and probabilities of occurrence of fecal scores of sheep fed levels of buffel grass hay and cactus pear.

**Score**	**Levels of Buffel Grass Hay, %**				***p*-Value**		
	**7.5 c**	**15 c**	**30 b**	**45 a**	**BH ^1^**	**P ^2^**	**BH x P ^3^**
	**%**						
0	2.6	3.8	2.8	100	0.0034	0.9991	0.9311
1	20.2	26.5	78.6	0			
2	70.7	63.2	12.1	0			
3	6.5	6.5	6.5	0			
	**Levels of Buffel Grass Hay, %**				**EP ^4^**	***p*-Value**	
	**7.5**	**15**	**30**	**45**		**L**	**Q**
DMI, kg/dia	1.05	1.38	1.30	1.07	0.235	0.6262	0.0101

^1^ BH = Buffel hay; ^2^ Collection period = collection days (1°, 7° and 21° day); ^3^ BH x P = Interaction between buffel grass hay levels and collection period. ^4^ Standard Error of the average. The means followed by different letters on the same line differ from each other by the Tukey test at 5% significance.

**Table 5:** Isolated effects for physiological parameters and interaction effect for belly temperature of sheep fed levels of buffel grass hay and cactus pear.

**Variables**	**Levels of Buffel Grass Hay (%)**				**Average**	**SEM**	***p*-Value**
	**7.5**	**15**	**30**	**45**			
T. ^1^ rectal (°C)	39.12	39.11	39.10	39.05	39.09	0.096	0.953
T. of the nape (°C)	35.99	36.56	36.55	37.24	36.58	0.439	0.263
T. of palette (°C)	34.29	33.94	34.28	34.86	34.34	0.325	0.257
T. of ham (°C)	33.46	32.24	34.00	34.48	33.54	0.814	0.246
RR ^2^ (mov.min^−1^)	25.72	25.55	25.22	24.89	25.35	1.296	0.496
Belly temperature (°C)	**Levels of Buffel Grass Hay %**	**Period/Day**		**SEM**	***p*-Value**		
		**1°**	**21°**		**BH ^3^**	**P**	**BH x P**
	7.5	36.24 Aa	35.40 Ba	0.243	0.393	0.275	0.031
	15	36.79 Aa	36.79 Aa				
	30	36.40 Aa	36.75 Aa				
	45	36.66 Aa	36.99 Aa				

Means followed by the same capital letter, in the columns, and lowercase in the rows, do not differ by Tukey’s test at 0.05 probability: SEM: standard error of the mean; ^3^ BH = Treatment; P = Period; BHxP = interaction effect; ^1^ T. = Temperature; ^2^ FR = Respiratory rate.

**Table 6:** Blood parameters of sheep fed levels of buffel grass hay and cactus pear.

Characteristic	Levels Buffel Grass Hay %	Period (Days)			Overall Average (BH)	F ^1^ Test *p*-Value			*p*-Value ^2^		
		1°	7°	21°		BH	P	Interaction(BH x P)	%BH		
									L	Q	C
Erythrocyte, mm^3^	7.5	6.73 ± 0.87	7.07 ± 0.87	8.55 ± 0.87	7.45 ± 0.75	0.4139	<0.0001	0.3695	0.5713	0.1404	0.5182
	15	5.84 ± 0.92	7.73 ± 0.92	9.86 ± 0.92	7.81 ± 0.79						
	30	9.04 ± 0.97	8.69 ± 0.95	9.96 ± 0.92	9.23 ± 0.81						
	45	7.68 ± 0.87	8.10 ± 0.87	9.27 ± 0.90	8.35 ± 0.76						
	Overall Average (P)	7.33 b ± 0.50	7.90 b ± 0.51	9.45 a ± 0.35							
Hemoglobin, g/dL	7.5	7.17 b ± 0.41	8.20 ab ± 0.43	9.24 a ± 0.43	8.20 ± 0.36	0.1888	<0.0001	<0.0001	0.2456	0.0628	0.8732
	15	5.91 b ± 0.43	7.98 a ± 0.43	9.07 a ± 0.45	7.65 ± 0.37						
	30	8.10 b ± 0.43	7.92 b ± 0.43	9.43 a ± 0.43	8.76 ± 0.36						
	45	8.62 b ± 0.43	8.63 b ± 0.41	9.02 b ± 0.43	8.26 ± 0.34						
	Overall Average (P)	7.45 ± 0.21	8.18 ± 0.21	9.19 ± 0.22							
Hematocrit, %	7.5	19.36 ± 2.45	20.78± 2.45	25.90 ± 2.45	23.28 ± 2.30	0.4201	<0.0001	0.1726	0.5145	0.1610	0.4890
	15	17.60 ± 2.59	22.58 ± 2.59	29.68 ± 2.59	27.23 ± 2.33						
	30	26.27 ± 2.67	24.91 ± 2.67	30.51 ± 2.59	24.46 ± 2.19						
	45	22.45 ± 2.45	23.03 ± 2.45	27.90 ± 2.51	22.01 ± 2.19						
	Overall Average (P)	21.42 b ± 1.27	22.83 b ± 1.27	28.50 a ± 1.27							
CHCM ^3^, g/dL	7.5	30.18 b ± 0.68	33.07 a ± 0.66	32.90 a ± 0.66	31.52 ± 0.55	0.4230	<0.0001	0.0092	0.2556	0.9691	0.2186
	15	31.12 b ± 0.69	32.51 b ± 0.69	30.92 b ± 0.69	31.21 ± 0.57						
	30	31.60 b ± 0.72	31.18 b ± 0.72	32.97 b ± 0.66	32.40 ± 0.53						
	45	32.97 b ± 0.66	33.16 b ± 0.66	31.08 b ± 0.68	32.05 ± 0.53						
	Overall Average (P)	31.27 ± 0.34	32.59 ± 0.34	31.52 ± 0.34							
Leukocytes, μL	7.5	7020.00 b ± 765.81	7510.00 b ± 765.81	7519.18 b ± 765.81	7349.73 ± 673.29	0.2666	0.0106	0.0090	0.4417	0.2737	0.1508
	15	5277.78 b ± 807.24	8011.11 a ± 807.24	7955.56 a ± 807.24	7081.48 ± 706.89						
	30	7266.67 b ± 807.24	7166.67 b ± 807.24	7122.22 b ± 807.24	7185.19 ± 706.89						
	45	8930.00 b ± 765.81	9488.74 b ± 780.49	7890.00 b ± 765.81	8769.58 ± 672.49						
	Overall Average (P)	7123.61 ± 393.40	8044.13 ± 395.20	7621.74 ± 395.96							
Segmented, μL	7.5	4339.70 b ± 471.60	4716.10 b ± 471.60	4361.00 b ± 471.60	4472.27 ± 409.58	0.5301	<0.0001	0.0083	0.5195	0.6331	0.2173
	15	3342.78 b ± 497.11	4791.34 ab ± 512.75	4891.44 a ± 497.11	4341.85 ± 433.77						
	30	3094.73 b ± 512.75	4849.11 b ± 497.11	4039.11 b ± 497.11	4194.32 ± 433.77						
	45	4749.42 ab ± 487.10	6095.64 a ± 487.10	4232.40 b ± 471.60	5025.82 ± 415.14						
	Overall Average (P)	4031.66 ± 246.18	5113.05 ± 246.18	4380.99 ± 242.26							
Eosinophiles, μL	7.5	118.60 b ± 26.66	182.90 b ± 24.44	155.63 b ± 26.66	152.38 ± 18.59	0.0393	0.0001	0.0066	0.4268	0.0156	0.5424
	15	97.22 b ± 26.82	173.96 b ± 28.11	205.79 b ± 28.26	158.99 ± 19.61				0.3693	0.6334	0.0492
	30	158.67 b ± 26.82	150.44 b ± 26.82	296.22 a ± 26.82	201.78 ± 19.19				0.0880	0.0028	0.2620
	45	190.21 b ± 26.66	231.80 b ± 25.44	234.25 b ± 26.66	218.75 ± 18.59						
	Overall Average (P)	141.17 ± 13.37	184.78 ± 13.23	222.97 ± 13.55							
Lymphocytes, μL	7.5	2289.50 ± 274.83	2052.74± 287.94	2362.10 ± 287.94	2234.78 ± 202.09	0.0343	0.0124	0.0779	0.2054	0.0507	0.0771
	15	1655.22 ± 289.70	2090.89 ± 289.70	2399.11 ± 289.70	2048.41 ± 208.81						
	30	2597.46 ± 305.94	1666.34 ± 305.94	2468.78 ± 289.70	2244.19 ± 214.99						
	45	3310.00 ± 274.83	2344.81 ± 287.93	2931.50 ± 274.83	2862.10 ± 200.15						
	Overall Average (P)	2463.05 a ± 143.31	2038.69 b ± 146.49	2540.37 a ± 142.81							
Monocytes, μL	7.5	217.85 b ± 49.38	392.30 ab ± 47.26	554.40 a ± 47.26	388.18 ± 35.64	0.4195	<0.0001	0.0024	0.1459	0.7624	0.4403
	15	183.33 b ± 49.82	423.00 a ± 49.82	379.28 ab ± 52.32	32,854 ± 37.61						
	30	308.44 b ± 49.82	322.00 b ± 49.82	384.78 b ± 49.82	338.41 ± 37.23						
	45	318.90 b ± 47.26	484.10 b ± 47.26	398.50 b ± 49.38	400.50 ± 35.64						
	Overall Average (P)	257.13 ± 24.54	405.35 ± 24.28	429.24 ± 24.86							
Platelets, μL	7.5	752,900 ± 71,317	699,300 ± 71,317	790,700 ± 71,317	747,633 ± 60,078	0.8455	0.2644	0.1051	0.7651	0.8834	0.4105
	15	706,667 ± 75,174	843,111 ± 75,174	836,444 ± 75,174	795,407 ± 63,328						
	30	726,222 ± 75,174	684,556 ± 75,174	840,349 ± 77,909	750,376 ± 63,694						
	45	857,100 ± 71,317	906,800 ± 71,317	768,700 ± 71,317	810,867 ± 60,078						
	Overall Average (P)	760,722 ± 36,636	758,422 ± 36,636	809,048 ± 36,991							
Total plasma Protein., g/dL	7.5	7.23 b ± 0.10	7.44 b ± 0.09	7.08 b ± 0.09	7.25 ± 0.06	0.5801	<0.0001	0.0142	0.3311	0.3211	0.8704
	15	7.03 b ± 0.10	7.52 a ± 0.10	6.98 b ± 0.10	7.18 ± 0.06						
	30	7.00 b ± 0.10	7.30 b ± 0.10	7.46 b ± 0.10	7.25 ± 0.06						
	45	7.19 b ± 0.09	7.48 b ± 0.10	7.21 b ± 0.09	7.29 ± 0.06						
	Overall Average (P)	7.11 ± 0.05	7.43 ± 0.05	7.18 ± 0.05							

^1^ BH = buffel grass hay; P = collection periods; ^2^ L = Linear, Q = quadratic and C = cubic; ^3^ CHCM = corpuscular hemoglobin concentration mean; the means followed by different letters on the same line differ from each other by the Tukey test at 5% significance.
